# The modulation of pancreatic lipase activity by alginates

**DOI:** 10.1016/j.foodchem.2013.09.075

**Published:** 2014-03-01

**Authors:** Matthew D. Wilcox, Iain A. Brownlee, J. Craig Richardson, Peter W. Dettmar, Jeffrey P. Pearson

**Affiliations:** a Institute for Cell and Molecular Biosciences, Medical School, Newcastle University, Framlington Place, Newcastle upon Tyne, Tyne and Wear NE2 4HH, UK; b Technostics Ltd., Daisy Building (2nd floor), Castle Hill Hospital, Castle Road, Hull HU16 5JQ, UK

**Keywords:** Alginate, Lipase, Inhibition, Obesity, Weight management

## Abstract

Alginates are comprised of mannuronic (M) and guluronic acid (G) and have been shown to inhibit enzyme activity. Pancreatic lipase is important in dietary triacylglycerol breakdown; reducing pancreatic lipase activity would reduce triacylglycerol breakdown resulting in lower amounts being absorbed by the body.

Lipase activity in the presence of biopolymers was assessed by enzymatic assay using natural and synthetic substrates. Alginate inhibited pancreatic lipase by a maximum of 72.2% (±4.1) with synthetic substrate (DGGR) and 58.0% (±9.7) with natural substrate. High-G alginates from *Laminaria hyperborea* seaweed inhibited pancreatic lipase to a significantly higher degree than High-M alginates from *Lessonia nigrescens*, showing that inhibition was related to alginate structure.

High-G alginates are effective inhibitors of pancreatic lipase and are used in the food industry at low levels. They could be included at higher levels in foods without altering organoleptic qualities, potentially reduce the uptake of dietary triacylglycerol aiding in weight management.

## Highlights

• Certain alginates are effective inhibitors of pancreatic lipase.
• The level of lipase inhibition by alginates is related to the structure of the polymer.
• High guluronate alginates achieve greater lipase inhibition than high mannuronate.
• Alginates have the potential to be a well-tolerated obesity treatment.

## 1 Introduction

In the western world, dietary fats can account for 40% of energy intake, with triacylglycerol (TAG) being the major component (Mu & Høy, 2004). Pancreatic lipase plays an important role in the hydrolysis of TAG with only 10–30% of hydrolysis occurring before the duodenal phase (Hamosh & Scow, 1973).

Pancreatic lipase has become a valid target in the treatment of obesity with the development of Tetrahydrolipstatin (orlistat®) (Drent & Vanderveen, 1993). Orlistat inhibits pancreatic lipase by covalently modifying the active site, reducing the hydrolysis of TAG (Borgstrom, 1988; Hadvary, Lengsfeld, & Wolfer, 1988). When taking orlistat, the level of ingested fat excreted in the faeces can be increased from 5% to 32% when compared to a placebo group (Zhi et al., 1994). In the UK, 98% of all prescriptions for the treatment of obesity in 2010 were for orlistat, the remaining 2% was for Sibutramine (withdrawn 2010) (The NHS Information Centre, 2012). Gastrointestinal side effects associated with orlistat treatment can often cause problems with patient compliance to the treatment regime, with below 50% compliance, even with pharmacist intervention (Gursoy, Erdogan, Cin, Cesur, & Baskal, 2006; Malone & Alger-Mayer, 2003). However, the gastrointestinal side effects of orlistat may be reduced if taken concomitantly with natural fibres, such as *Psyllium* mucilloid (Cavaliere, Floriano, & Medeiros-Neto, 2001).

Alginates are dietary fibres consisting of a linear polymer containing two epimers of uronic acid, mannuronic (M) and guluronic acid (G) (Haug & Smidsrod, 1967). Alginates can be extracted from the cell walls of brown seaweed or from certain bacteria. For example, alginates are the major constituents of the vegetative capsule of the rigid and desiccation resistant walls of metabolically dormant cysts in the soil bacteria *Azotobacter vinelandii* (Haug & Smidsrod, 1967).

Certain polymers have been shown to have an effect on triacylglycerol hydrolysis, such as chitin–chitosan mixtures and polydextrose with diethylaminoethyl groups attached (Han, Kimura, & Okuda, 1999; Tsujita et al., 2007). Both of these polymers potentially affect the substrate and the interface between substrate and enzyme.

Alginates have previously been shown to have an inhibitory effect on gastrointestinal enzymes. In 2000 Sunderland et al., showed that alginates reduced the activity of pepsin by an average of 52% *in vitro* (Sunderland, Dettmar, & Pearson, 2000). The work identified the characteristics of alginates that correlated with the level of pepsin inhibition (Sunderland, Dettmar, & Pearson, 2000). The molecular weight of the alginate was key to the level of pepsin inhibition achievable (Strugala, Kennington, Campbell, Skjak-Braek, & Dettmar, 2005; Sunderland et al., 2000).

The previously shown bioactivity of alginate can be altered by both sugar residue composition and molecular weight. The use of the epimerase enzymes allow alginates to be modified to a specifically desired ratio of M and G residues as well as the order of residues, therefore allowing designer alginates to be produced, which would be vital to the understanding of which characteristics of an alginate are important in a biological system.

Here we hypothesise that pancreatic lipase activity can be inhibited by alginates and that the extent can be modulated to a different degree dependent on the structural characteristics of alginate used. Well characterised alginates from both sources (bacteria and seaweed) were used in this study, including alginates that were enzymatically modified.

## 2 Materials and methods

### 2.1 Materials

All alginate samples were kindly provided by Technostics Limited (Hull, UK) (Table 1). The bile acids (deoxycholate sodium salt and taurodeoxycholate sodium salt) were both purchased from Fluka (Buchs, Switzerland). The lipase, colipase and orlistat (tetrahydrolipstatin), tris(hydroxymethyl)-methylamine, 1,2 Di-o-lauryl-rac-glycero-3-(glutaric acid 6-methyl resorufin ester) (DGGR), sodium acetate, calcium chloride and acetone were all purchased from Sigma–Aldrich (Poole, UK). The olive oil was purchased from a local supermarket (Cooperative Foods, UK) and the aluminium oxide was purchased from Fisher Scientific (Loughborough, UK).

### 2.2 Lipase activity assay using DGGR as the substrate

The lipase activity assay was a modified version of the method developed by Panteghini, Bonora, and Pagani (2001). The assay was comprised of three solutions; solution 1, solution 2 and the lipase solution. Solution 1; Tris buffer (50 mmol/l, pH 8.4 at 23 °C), 1 mg/l of colipase and 1.8 mM deoxycholate sodium salt. Solution 2; acetate buffer (18 mmol/l, pH 4.0 at 23 °C) 72 mM taurodeoxycholate sodium salt, 0.1 mM calcium chloride and 0.24 mM DGGR. Solution 2 was mixed with a magnetic stirrer at 500 rpm and 4 °C overnight. The lipase solution contains 1 g/l of porcine pancreatic lipase in deionised water, where 1 mg contains 60 U of lipase activity (where one unit will hydrolyse 1.0 microequivalent of fatty acid from a triglyceride in one hour at pH 7.4 using triacetin).

A 4 mg/ml stock solution of each polymer was prepared by slowly adding lyophilised biopolymer to the vortex formed by vigorously stirring solution 1 on a magnetic stirrer. The resulting stock solution (4 mg/ml) was then further diluted with solution 1 to achieve 1 and 0.25 mg/ml samples. This achieved a concentration of 3.43, 0.86 and 0.21 mg/ml, respectively in the reaction mixture. Two controls were used in the assay, an inhibition control (100% inhibition) and a lipase control (0% inhibition). The inhibition control contained 0.025 mg/ml orlistat added to solution 1 and the lipase control was the standard reaction with no inhibitors or biopolymers. All solutions were stored at 4 °C for up to 24 h.

The assay was set up over two 96 well microplates. The first contained 15 μl of solution 2 in every well. The second plate contained 180 μl of solution 1, or a concentration of biopolymer in solution 1. 12 μl of lipase solution was also added to every well (12 μl of deionised water for reagent blank well) on the second plate. The two plates were incubated for 1 h at 37 °C then 160 μl of the second plate was added to the first plate to initiate the reaction.

To calculate the percentage of lipase inhibition, the reagent blanks were subtracted from the corresponding controls or samples and the following formula was applied:

Percentage of Lipase Inhibition=1-((Polymer Sample-Inhibition Control)/(Lipase Control-Inhibition Control))×100

### 2.3 Lipase activity assay using olive oil as the substrate

The olive oil assay system uses a modified version of the method of Vogel and Zieve (1963). The turbidimetric method measures the reduction in turbidity that occurs following the breakdown of TAGs to free fatty acids by lipase. Olive oil, with a specific viscosity of 72.5 (±10), (specific viscosity used here is unitless as it is derived from a ratio of the oil to that of water) was used throughout the series of experiments. The olive oil was passed through aluminium oxide (80 × 15 mm deep in a glass chromatography column) to remove free fatty acids. 10.0 g of the olive oil free from fatty acids was made up to 100 ml with acetone giving a 10% solution. This is turn was diluted 1 in 10 with acetone to achieve a 1% olive oil stock solution. The stock solution was stored at 4 °C for up to four weeks and was used over the entire series of experiments.

For use in the assay, an olive oil substrate solution was prepared by adding 4 ml of 1% stock olive oil solution to a heated solution (70 °C) of 100 ml 0.05 M Tris buffer at pH 8.3 containing 0.35% sodium deoxycholate. This solution was maintained at 70 °C and homogenised for 10 min. Once the froth had settled and the solution had returned to room temperature, the substrate solution could be used in the assay for up to 6 h.

The enzyme solution contained 1.29 mg/ml lipase and 18 μg/ml colipase in deionised water. Orlistat was added (0.025 mg/ml) to the enzyme solution as an inhibition control. Biopolymers were added to the freshly prepared substrate solution containing the olive oil to give 3.6, 0.9 and 0.23 mg/ml.

The samples were incubated at 37 °C for 15 min. After the incubation the substrate solution was added to the solution containing the enzyme solution or deionised water. The assay was maintained at 37 °C and read every 5 min at 405 nm for 35 min.

To calculate the percentage of lipase inhibition, the blanks were subtracted from their respective controls and the following equation was applied

Percentage of lipase inhibition=1-((Inhibition Control-Polymer Sample)/(Inhibition Control-Lipase Control))×100

### 2.4 Statistical analysis

All data were analysed using GraphPad Prism 4 statistical software. The comparison of inhibition levels with seaweed species was made by using a two way ANOVA. Comparison of carbohydrate content and inhibition was made after plotting the data and assessing correlation using a nonparametric Spearman test. For initial characterisation of the assay systems (DGGR and olive oil) three replicates were used. The analysis between the level of inhibition by alginates from two seaweed sources was tested with a one way ANOVA with Tukey post test. All subsequent measurements used six replicates. The number of replicates is shown in each figure legend.

## 3 Results

**Fig. 1:**
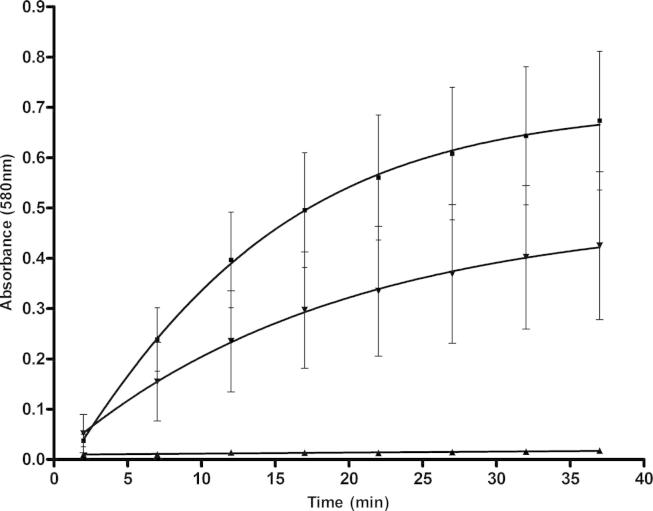
Lipase activity using the DGGR assay measured in absorbance over time. ■ – Lipase control (lipase plus substrate), ▾ – alginate SF200 at 3.43 mg/ml (as an example) and ▴ – inhibition control 0.025 mg/ml orlistat. The error bars show the standard error of the mean of 3 replicates. All enzyme inhibition is calculated with data from 12 min as the reaction is still close to a linear phase.

Using DGGR as a substrate, the activity of lipase could be measured by the increase in absorbance (Panteghini et al., 2001). As expected, there was a marked change in the absorbance over time for the negative control (lipase plus substrate) illustrating the maximum rate of reaction (Fig. 1), whereas, for the inhibition control (Orlistat (0.025 mg/ml)) there was minimal change in absorbance over time. Fig. 1 also shows that alginate could inhibit the activity of lipase. To compare the inhibition of a range of alginates, the absorbance at 12 min reaction time was chosen. This time point was used because the reaction was still close to the linear phase.

**Fig. 2:**
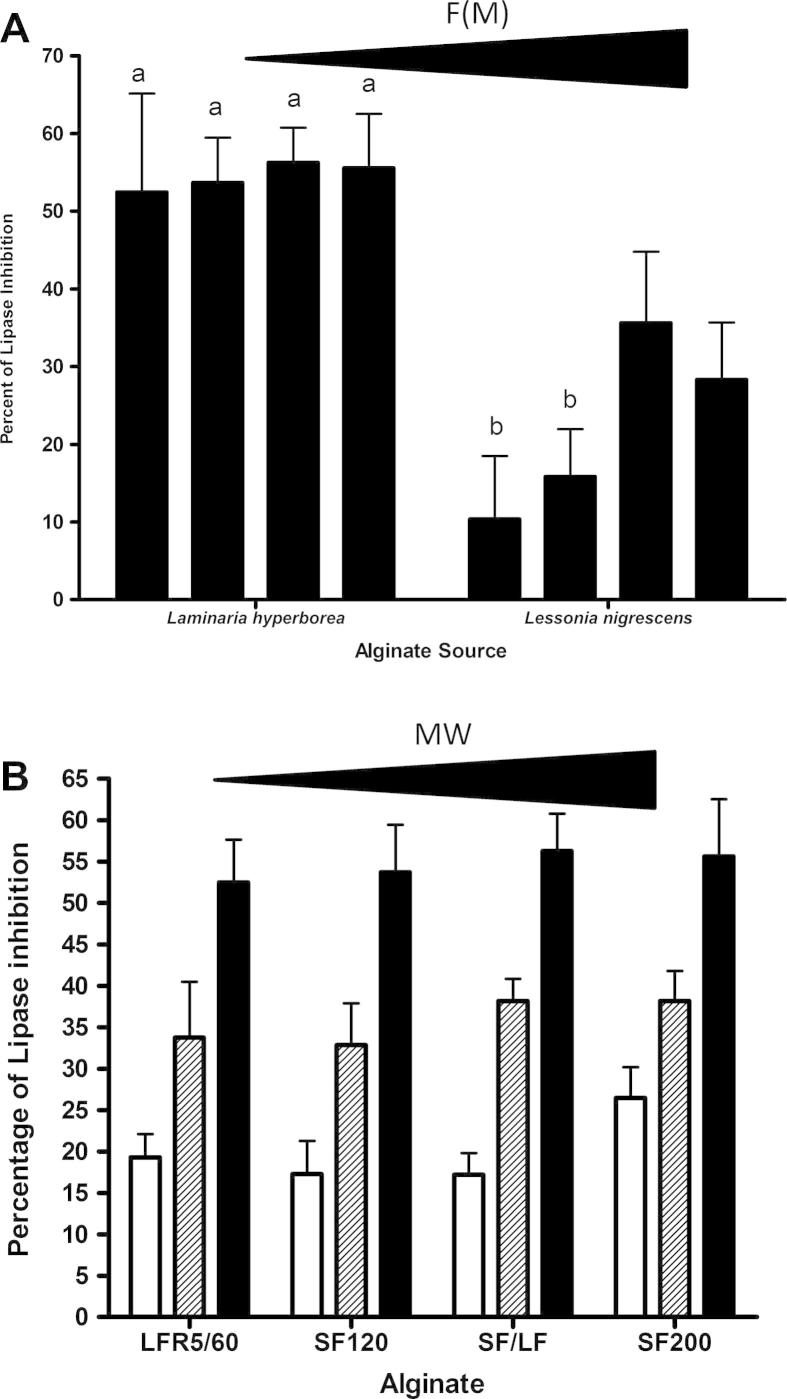
(A) Inhibition of lipase caused by *Laminaria hyperborea* and *Lessonia nigrescens* alginates (DGGR substrate). Four alginates of differing molecular weight are shown for *Laminaria hyperborea* and *Lessonia nigrescens* as their respective source. Inhibition of lipase shown in this figure is for 3.43 mg/ml of alginate. The alginates from *Laminaria hyperborea* species are (from left to right) LFR5/60, SF120, SF/LF, and SF200, full characteristics can be found in Table 1. The *Lessonia nigrescens* seaweed alginates are (from left to right) LF10L, LF120L, SF60 and H120L, again full details can also be found in Table 1. Error bars shown are the standard error of the mean of six replicates. *Laminaria hyperborea* alginates inhibit lipase to significantly higher degree than *Lessonia nigrescens* alginates (*p* = 0.0015). Bars annotated with letter 'a' are significantly different to those with letter 'b'. (B) Concentration dependent inhibition of lipase by the four alginates from *Laminaria* genus of seaweeds (DGGR substrate). □ – 0.21 mg/ml, ▨ – 0.86 mg/ml and ■ – 3.43 mg/ml. The error bars are the standard error of the mean of six replicates.

Fig. 2A shows that there was a significant difference in the level of inhibition depending on the seaweed source of the alginate. The alginates extracted from *Laminaria hyperborea* seaweed inhibited pancreatic lipase to a significantly higher degree (two way ANOVA, *p* = 0.0015) than the alginates extracted from *Lessonia nigrescens*.

A dose dependent inhibition was seen for both sets of seaweed alginates. Fig. 2B shows that for *Laminaria hyperborea* alginate the percentage of lipase inhibition increased with increasing concentration. For LFR5/60, there was a 75% relative increase in inhibition when the alginate concentration was increased fourfold from 0.21 mg/ml to 0.86 mg/ml, and a 56% increase from 0.86 mg/ml to 3.43 mg/ml. Similarly, the increases for alginates SF120 and SF/LF were 90% and 122%, respectively when the alginate concentration was increased from 0.21 to 0.86 mg/ml, and again 64% and 47%, respectively increased to 3.43 mg/ml. The alginate SF200 level of inhibition increased 44% and 46% when the alginate concentration increased from 0.21 to 0.86 and then 3.43 mg/ml.

As seen in Fig. 2, not all alginates inhibited lipase to the same extent, even those from the same genus of seaweed. To understand why the levels differed, the compositional characteristics of the various alginates were correlated with the level of lipase inhibition (Table 2). Statistical significant positive correlations were found between levels of inhibition and increasing guluronate content (F(G)), the fraction of guluronate dimers (F(GG)), the fraction of guluronate trimers (F(GGG)) and the number of guluronate blocks greater than one in the alginate polymer (N(G > 1)). Surprisingly, the reciprocal correlations with mannuronate levels were not always significant. Only F(M) and F(MG) were statistically significant negative correlations. The range of molecular weight tested in this study also did not have an effect on lipase inhibition (see Table 2).

**Fig. 3:**
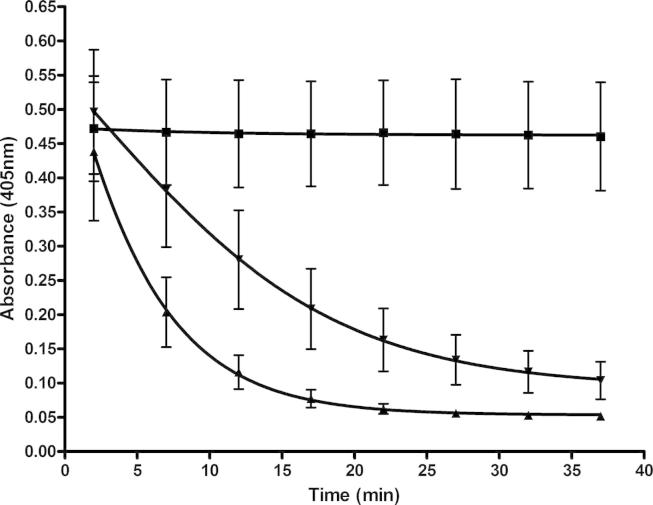
The absorbance change (at 405 nm) over time as a measure of lipase activity (olive oil substrate). ▴ – Lipase control (lipase plus substrate), ▾ – alginate SF200 at 3.43 mg/ml (as an example) and ■ – inhibition control 0.025 mg/ml orlistat. The error bars show the standard error of the mean of three replicates. All enzyme inhibition was calculated with data from 12 min.

**Fig. 4:**
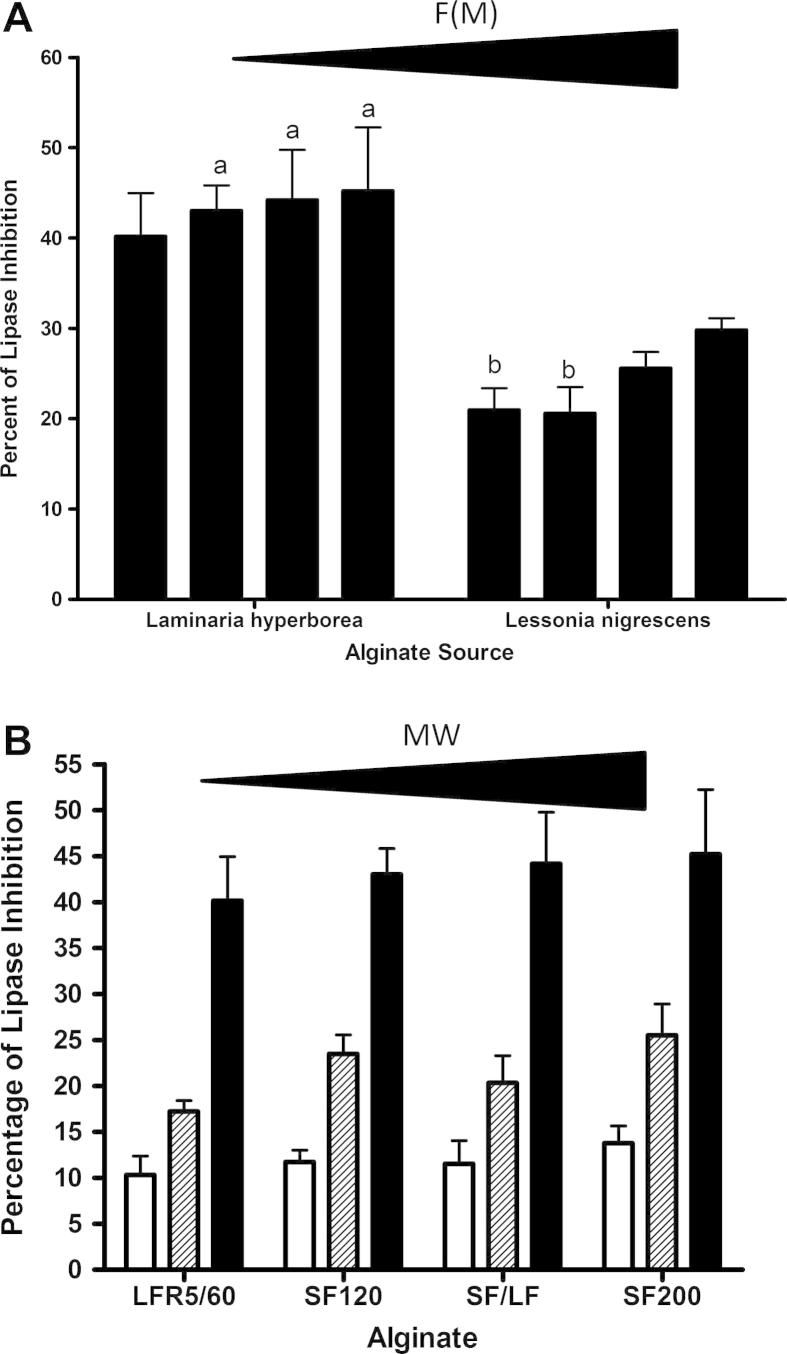
(A) Comparison of lipase inhibition, using olive oil as a substrate, by alginates extracted from *Laminaria* and *Lessonia* seaweeds. Four alginates of increasing molecular weight from left to right are shown for both *Laminaria* and *Lessonia* as their respective source. Inhibition of lipase shown in this figure is caused by 3.43 mg/ml of alginate. There was a statistical difference between the two species of seaweed with p values of less than 0.001. Error bars shown are the standard error of the mean of three replicates. Bars annotated with letter 'a' are significantly different to those with letter 'b'. (B) Concentration dependent inhibition of lipase by the four alginates from *Laminaria hyperborea* genus of seaweeds (olive oil substrate). □ – 0.21 mg/ml, ▨ – 0.86 mg/ml and ■ – 3.43 mg/ml. The error bars are the standard error of the mean of three replicates. The molecular weight of the biopolymers increases from left to right (LFR5/60 to SF200).

To understand the effects of alginates on lipase activity further we decided to investigate the relationships using a different assay system that used a natural substrate, olive oil. Lipase activity was measured by changes in turbidity of an olive oil solution due to the breakdown of triacylglycerol to free fatty acids and monoacylglycerol. As seen with the previous assay system, alginate again inhibited lipase activity (Fig. 3) and the levels of inhibition varied depending on the alginate source (Fig. 4A). Although the levels of inhibition with an olive oil substrate were lower than that shown with the synthetic substrate, they were not significantly different.

However, the difference between the two species of alginates was statistically significantly different (Fig. 4A) using olive oil as the substrate. Alginates extracted from the seaweed species *Laminaria hyperborea* inhibited pancreatic lipase to a significantly higher degree than alginates extracted from *Lessonia nigrescens* (Fig. 4A). The difference between the two alginate sources was apparent at all concentration of alginate tested; at 3.43, 0.86 and 0.21 mg/ml with a *p* value of less than 0.001 for 3.43 and 0.86 mg/ml, and a *p* value of 0.012 for the 0.21 mg/ml.

There was less variation between the level of inhibition by the *Lessonia nigrescens* alginates when comparing the two substrates; 10.4% (±8.1) – 35.6% (±22.4) for DGGR (Fig. 2A) compared to 21.0% (±4.1) – 29.8% (±2.3) for olive oil (Fig. 4A). The same statistical difference was observed with the *Laminaria hyperborea* alginates showing an overall higher level of inhibition compared to the *Lessonia nigrescens* alginates, whichever substrate was used.

A dose dependent relationship for the *Laminaria hyperborea* alginate was again demonstrated with olive oil as the substrate. Fig. 4B shows that decreasing the concentration from 3.43 to 0.86 and then to 0.21 mg/ml of alginate in the reaction mixture decreases the level of lipase inhibition. The same is also true for alginates extracted from *Lessonia nigrescens* species seaweed (data not shown). There was an increase of 1.7-fold and 2.3-fold in the level of inhibition when the concentration of LFR5/60 alginate was increased from 0.21 mg/ml to 0.86 mg/ml and from 0.86 mg/ml to 3.43 mg/ml. This was also similar for the other three *Laminaria hyperborea* alginates. When there was a fourfold increase in the alginate concentration from 0.21 mg/ml to 0.86 mg/ml, there was an increase in the level of inhibition observed, 100.0%, 76.4%, and 85.0% increase for SF120, SF/LF, and SF200, respectively. When the concentrations of the same alginates were increased fourfold (3.43 mg/ml), again the level of inhibition increased 83.2%, 117.2%, and 77.4%, respectively for alginates SF120, SF/LF, and SF200.

The ability of alginates to inhibit lipase was related to structural/compositional characteristics using olive oil as a substrate as seen in Table 2. Unlike the DGGR substrate, the absolute amount of alginate that was guluronate was not significant but as before the F(GG), F(GGG), and N(G > 1) still correlated significantly with lipase inhibition (Table 2).

## 4 Discussion

The adapted methods of Panteghini et al. (2001) and Vogel and Zieve (1963) are both effective for *in vitro* determination of pancreatic lipase activity. There are drawbacks and advantages with both methods used in this paper. DGGR is a synthetic substrate whereas olive oil is a natural substrate, but being a natural substrate olive oil is a mixture of different triacylglycerol with varying acyl chain length, which will have differing affinity for the enzyme (Jemel, Fendri, Gargouri, & Bezzine, 2009; Rogalska, Ransac, & Verger, 1990). The enzyme would also have to act on the substrate twice for there to be a detectable change in the optical density (OD), as diacylglycerol would not be solubilised and therefore not reduce the OD. This could explain the lower levels of inhibition seen using the olive oil as a substrate compared to the DGGR substrate which is only cleaved once.

The two methods show relatively large error bars, which can be explained to some extent by the solubility of the substrate. This varied between the replicates however for each experiment the same substrate preparation has been used for the positive control, negative control and the inhibition study.

Both methods showed that alginates are effective inhibitors of pancreatic lipase, depending upon the structure. Alginates with a high G block content can inhibit lipase to a much greater extent than alginates with high M block content. Therefore, it is possible to modulate the activity of pancreatic lipase to a varying degree depending upon the alginates used.

Molecular weight of the alginates was not a determining factor of inhibition and neither was viscosity as one of the best inhibitors, LFR5/60, has a viscosity of 6 mPas compared to a poor inhibitor, LF120L, which has a viscosity of 121 mPas (for 1% solution in phosphate buffered saline).

There are several potential mechanisms for inhibition of lipase by alginate. Alginates have the potential to interact with both the substrate and the enzyme itself. Alginates with a high G block content are known to interact with glycoprotein, specifically mucin measured by rheological assessment across a range of mucin: alginate ratios (Taylor, Draget, Pearson, & Smidsrød, 2005; Taylor, Pearson, Draget, Dettmar, & Smidsrød, 2005). It was hypothesised that alginate can interact with specific sites along the protein section of the glycoprotein, cross linking several mucin molecules together forming a gel (Taylor, Draget, Pearson, & Smidsrød, 2005). The G block content of the alginate was also key in the mucin interaction, as alginates with high mannuronate content would not interact and cross link the mucin molecules. Therefore showing that alginate can interact with protein and that G blocks are important for this interaction.

It is also possible that alginates may associate with the oil/water interface potentially reducing the access that lipase has to the interface. This is believed to be the mode of action of other potential inhibitors of pancreatic lipase such as chitosan, DEAE-Sephadex and DEAE polydextrose, all of which however are cationic whereas alginate is anionic (Han et al., 1999; Tsujita et al., 2007). DEAE-Sephadex and DEAE polydextrose have multiple diethylaminoethyl groups and can reduce the activity of lipase *in vitro*, which was dependent upon the degree of DEAE substitution. Increasing the degree of substitution of DEAE-polydextrose decreased the concentration needed to inhibit 50% activity. The concentration of polymer for 50% inhibition was 1.44, 16.9, 618, and >1000 μg/ml when the substitution degree was 1.09, 0.18, 0.079 and 0.048, respectively. The activity returns however when the substrate was emulsified with TritonX-100, a commonly used (uncharged) emulsifier (Han et al., 1999; Tsujita et al., 2007), therefore potentially outcompeting DEAE-Sephadex and polydextrose for space at the interface.

Isaksson, Lundquist, and Ihse (1982) showed that pectins with low esterification could inhibit pancreatic lipase in both a buffered system and in human pancreatic juice, with a more pronounced effect in the pancreatic juice (Isaksson et al., 1982). At higher levels of esterification (53%), pectin has also been shown to match the levels of inhibition achievable to that of the commercially available drug orlistat, 82% inhibition against 88% inhibition for that of orlistat (Kumar & Chauhan, 2010). The authors go onto suggest that pectin does not just interact with the substrate as is suspected to be the case with chitosan, but can actually complex with the enzyme and potentially protonate serine and histidine in the active site of the enzyme (Kumar & Chauhan, 2010). There was little detail regarding the units of activity of the lipase or the concentrations of substrate used in their experiment. However, in recent tests within our laboratory, commercially available pectin with a similar degree of esterification (60% compared to 53%) could only achieve 11.1% inhibition with olive oil as the substrate (3.8 mg/ml pectin against 3.4 U/ml enzyme – data not shown).

It is believed that the carboxyl groups of the pectin are involved in the protonation of the active site residues (Kumar & Chauhan, 2010). The carboxyl groups of pectin are where methyl groups are added via ester bonds, and increasing the level of esterification lowers the number of carboxyl groups. This therefore may explain why pectins with a higher level of esterification have a lower effect on lipase activity (Isaksson et al., 1982). The carboxyl groups in G block structures of alginate are in similar positions to that of the backbone of pectin molecules, which is how both bind calcium.

Dietary fibres would be advantageous in the treatment of obesity over conventional pharmaceutical lipase inhibitors, such as orlistat, as high fibre products can potentially reduce or eliminate the gastrointestinal side effects seen with orlistat treatment (Cavaliere et al., 2001). Dietary fibres, such as pectin and alginate show evidence of inhibiting lipase and could be incorporated into a wide variety of different vehicles for delivery. Alginates may be a more desirable candidate to take forward as an obesity treatment as they demonstrated a far superior lipase inhibiting capacity and can easily be modified enzymatically to produce the desired characteristics.

Alginates have previously been shown to increase fatty acid excretion in ileostomy patients, believed to be a result of the entrapment with the alginate matrix (Sandberg et al., 1994). The increase in fatty acid excretion may now be explained by the alginates capacity to inhibit lipase and therefore reduce the amount absorbed by the body.

Specific alginates are effective inhibitors of pancreatic lipase and have been used in the food and pharmaceutical industry for many years. The inclusion of an alginate into foods (without altering taste or acceptability) has the potential to reduce the intake of dietary triacylglycerol and could greatly help in weight management.

## Conflicts of interest

None of the authors have declared a conflict of interest.

## Figures and Tables

**Supplementary Fig. 1:**
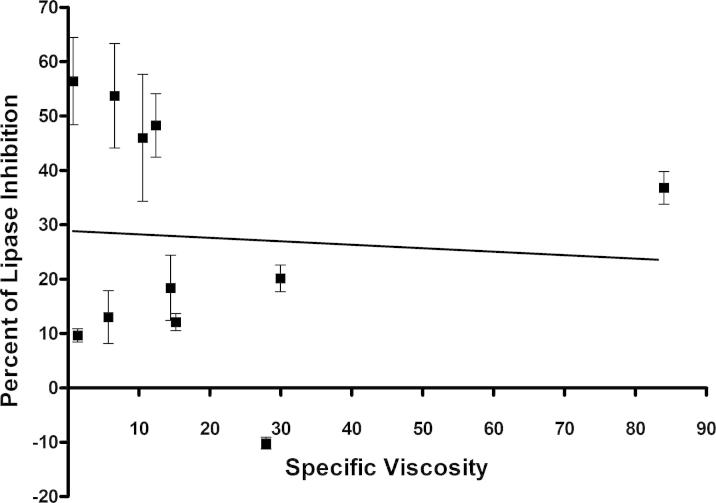
Correlation of the specific viscosity of alginate against lipase inhibition in the reaction mixture. The specific viscosity of each reaction mixture for the lipase activity assay containing 3.43 mg/ml was measures with a Contraves low shear 30. The error bars are the standard error of the mean of three replicated. The line of best fit is to indicate the direction of correlation, if any. A correlation analysis indicated a Sprearman *r* value of −0.27.

**Table 1:** The alginates used in this study with some of their characteristics.

	Alginate source	Molecular weight	F(G)	F(M)	N(G > 1)	Lipase inhibition (%)
1	Mannuronan (treated with epimerase)	423,767	0.8	0.2	44	72.2
2	Mannuronan (treated with epimerase)	262,400	0.7	0.3	7	41.5
3	Mannuronan (treated with epimerase)	374,800	0.68	0.32	22	59.1
4	Seaweed (treated with epimerase)	251,220	0.68	0.32	9	56.1
5	Seaweed *L. hyperborea* SF200	387,000	0.68	0.322	16.7	55.6
6	Seaweed (treated with epimerase)	226,550	0.67	0.33	9	62.4
7	Seaweed *L. hyperborea* SF120	195,000	0.664	0.336	9.6	53.7
8	Seaweed *L. hyperborea* SF/LF	295,000	0.66	0.336	13.8	56.3
9	Seaweed *L. hyperborea* LFR5/60	34,700	0.633	0.367	9.9	52.5
10	Mannuronan (treated with epimerase)	211,833	0.62	0.38	8	60.2
11	Mannuronan (treated with epimerase)	202,000	0.62	0.38	5	58.4
12	Mannuronan (treated with epimerase)	194,833	0.54	0.46	4	60.8
13	Mannuronan (treated with epimerase)	436,767	0.52	0.48	11	62.4
14	Seaweed *Laminaria hyperborea*	260,600	0.49	0.51	9	50.2
15	Mannuronan (treated with epimerase)	456,733	0.47	0.53	0	-2.3
16	Seaweed *L. nigrescens* LF10L	75,000	0.45	0.553	4.4	10.4
17	Seaweed *L. nigrescens* H120L	397,000	0.45	0.551	5.9	28.3
18	Mannuronan (treated with epimerase)	442,367	0.44	0.56	8	64.5
19	Seaweed *L. nigrescens* LF120L	221,000	0.424	0.576	4.7	15.8
20	Seaweed *L. nigrescens* SF60	325,000	0.411	0.589	3.3	35.6
21	Seaweed *Durvillea potatorum*	241,067	0.35	0.65	4	33.5
22	Mannuronan (treated with epimerase)	438,733	0.17	0.83	0	38.5
23	Bacterial mannuronan	584,400	0	1	0	53.2

Molecular weight is in Daltons, F(G) and F(M) are the fraction of guluronate and mannuronate residues present in the polymer determined by NMR. N(G > 1) is the number of consecutive guluronate residues in the alginate polymer.

**Table 2:** The Spearman’s correlation coefficient with *p* values of the correlation analysis of alginate characteristics against percentage of lipase inhibition with both DGGR and olive oil as the substrate.

Alginate characteristic	DGGR			Olive oil		
	Corr.	*p* Value	Signif.	Corr.	*p* Value	Signif.
F(G)	0.502	0.015	∗	0.380	0.074	n/s
F(GG)	0.583	0.004	∗∗	0.514	0.012	∗
F(GGG)	0.578	0.004	∗∗	0.593	0.003	∗∗

F(M)	−0.506	0.014	∗	−0.380	0.074	n/s
F(MM)	−0.336	0.117	n/s	0.0870	0.693	n/s
F(MG)	−0.505	0.014	∗	−0.806	<0.0001	∗∗∗
F(MGM)	−0.494	0.017	∗	−0.684	0.0003	∗∗
F(MGG)	−0.003	0.988	n/s	−0.377	0.076	n/s
N(G > 1)	0.586	0.003	∗∗	0.625	0.001	∗∗
Molecular weight	0.044	0.840	n/s	0.261	0.229	n/s

The characteristics of alginates, Spearman’s correlation coefficient, *r_s_* (Corr), *p* value and whether the correlation was statistically significant (Signif.) or not (significance is taken as *p* values below 0.05). F(G) is the fraction of guluronate in the alginate, F(GG) is the fraction of guluronate dimers, F(GGG) is the fraction of guluronate trimers. F(M), F(MM) and F(MMM) are the fraction of mannuronate, its dimers and trimers in the alginate. F(MG) and F(MGM) are the fractions of alternating dimers and trimers with F(MGG) being the fraction of uronate trimer consisting of mannuronate and two guluronate residues specifically in that order. N(G > 1) is the number of guluronate blocks greater than one in the alginate polymer. *p* Values < 0.05 are represented by ∗, <0.005 are represent by ∗∗ and <0.001 are represented by ∗∗∗.
